# Characterization, protein modeling, and molecular docking of factor C from Indonesian horseshoe crab (*Tachypleus gigas*)

**DOI:** 10.1186/s43141-023-00496-8

**Published:** 2023-04-11

**Authors:** Apon Zaenal Mustopa, Ayu Fitri Izaki, Suharsono Suharsono, Fatimah Fatimah, Fauziyah Fauziyah, Rahmi Damarani, Arwansyah Arwansyah, Setyanto Tri Wahyudi, Siswi Sekar Sari, Rozirwan Rozirwan, Zubaidi Bachtiar

**Affiliations:** 1 Research Center for Genetic Engineering, National Research and Innovation Agency (BRIN), Bogor, 16911 Indonesia; 2 Graduate School of Biotechnology, IPB University, Bogor, Indonesia; 3 School of Biotechnology, IPB University, Bogor, Indonesia; 4 Marine Science Study Program, Faculty of Mathematics and Natural Science, University of Sriwijaya, Palembang, Indonesia; 5 Department of Chemistry Education, Faculty of Teacher Training and Education, Tadulako University, Palu, Indonesia; 6 Department of Physics, Faculty of Mathematics and Natural Sciences, IPB University, Bogor, 16680 Indonesia

**Keywords:** Amebocyte lysate, Endotoxin, Factor C, *Tachypleus gigas*, Molecular dynamic

## Abstract

**Background:**

Horseshoe crab (*Tachypleus gigas*) amebocytes are useful biomedical components for endotoxin detection, and their growing needs for biomedical purposes cause the horseshoe crab population to decline. Factor C synthesis via genetic engineering offers a solution to replace natural horseshoe crab’s factor C and prevent its excessive harvest from nature. In response to these concerns, this study aimed to characterize the amebocyte lysates and factor C protein modeling of *T. gigas* originated from Banyuasin South Sumatra Estuary.

**Methods and results:**

Sampling of *T. gigas* was carried out in Banyuasin South Sumatra Estuary, Indonesia. The endotoxin test or TAL (*Tachypleus* amebocyte lysates) assay was performed using gel coagulation method. Protein characterization of protease enzyme was conducted by protease activity, SDS-PAGE, and zymogram analysis. The cDNA of mitochondrial COI gene was amplified for molecular identification followed by cDNA cloning of factor C. Protein modeling was investigated by molecular docking and molecular dynamic (MD) simulation. Endotoxin test results showed that TAL-35 had endotoxin sensitivity in a range of 0.0156–1 EU/ml, while TAL 36 had a sensitivity between 00,625 and 1 EU/ml. *T. gigas* amebocytes have protease activity in molecular mass sizes less than 60 kDa, with 367 U/ml for TAL 35 and 430 U/ml for TAL 36. The molecular identification revealed 98.68% identity similarity to *T. gigas*. The docking results suggested three ligands; i.e., diphosphoryl lipid A, core lipid A, and Kdo2 lipid A can be activators of the factor C protein by binding to the region of the receptor to form a ligand-receptor complex.

**Conclusions:**

Endotoxins can be detected using horseshoe crab amebocytes. The presence of proteases is considered responsible for this ability, as evidenced by casein zymogram results. According to docking and MD analysis, we found that lipopolysaccharides (LPS) participate to the binding site of factor C.

## Background

Endotoxins, also known as lipopolysaccharides (LPS), are components of the outer membrane of gram-negative bacteria that are released into the bloodstream when bacteria undergo lysis or cell death. It is the most common pyrogens found in parenteral drugs and medical devices. Their presence in the bloodstream can result in a septic reaction and induce symptoms such as fever, hypotension, nausea, chills, and shock. Endotoxin concentrations above a certain threshold is causing serious complications such as disseminated intravascular coagulation (DIC), endotoxin shock, and adult respiratory distress syndrome (ARDS) [1]. Therefore, biomedical devices and pharmaceutical products must be free of pyrogens. Endotoxins are also a challenge for industrial sterilization. The Food and Drug Administration (FDA) approved the endotoxin contamination level to be at 0.5 endotoxin U/ml for medical devices or 20 EU/device for medical products that come into contact with the cardiovascular and lymphatic systems [2].

Limulus amebocyte lysate (LAL) test is considered as the most common endotoxin test. The test was carried out based on the interaction between LPS and an amebocyte cell protein (freezing protein/LAL) derived from the blood of horseshoe crabs. Those aqueous extract-clottable protein can be obtained after lysis of blood cells (amebocytes). Amebocytes lysate from *Carcinoscorpius rotundicauda* is called CAL, while amebocytes lysate from genus *Tachypleus* is called TAL. Interaction between endotoxin and LAL initiates a series of enzymatic reactions that activates pathways to produce a minimum of three serine protease zymogens (factor C, factor B, and pro-clotting enzymes). The activated pathway converts amebocyte coagulogen (a clottable protein such as invertebrate fibrinogen) to form a coaguline gel [3].

Because of the unique ability of horseshoe crab amebocyte lysates to detect endotoxins, commercial demand from pharmaceutical and biomedical companies to confirm the safety of drugs, food, and medical devices has increased. The Atlantic States Marine Fisheries Commission (ASMFC) assumes that horseshoe crabs have a mortality ratio of at least 15–30%. The death of horseshoe crab can be caused by taking blood for biomedical purposes [4]. Horseshoe crab blood sampling was carried out as much as 10–25% of the total body weight [5]. If the blood is taken up to 40% of the total body weight, 15.4% of horseshoe crabs will experience stress to death [6]. Collecting horseshoe crab blood may seem like a simple process. However, there will be significant risks to horseshoe crabs in several processes from transportation, storage of horseshoe crabs to blood collection. Furthermore, it does not account additional stress or adverse behavioral changes after animals are returned to the sea, nor does it consider the impact on offspring populations of horseshoe crabs whose spawning is impaired as a result of blood collection [7].

The negative impacts of using horseshoe crab blood for medical purposes have prompted many researchers to look for long-term alternatives to save horseshoe crab, such as the following: (i) cultivation of horseshoe crab for blood supply [8], (ii) culture of amebocyte tissue that produces LAL [9], and (iii) genetic engineering to produce protein lysates such as factor C. Recombinant Factor C (rFC) is possible to be biotechnologically engineered and considered as a protein at high purity and low inter-lot variability. The rFC testing allows detection of high endotoxin specificity. As a result, rFC-based assays may improve sensitivity and accuracy of traditional bacterial endotoxin detection. Furthermore, rFC recombinant proteins seem to be more feasible to be produced long term and in unlimited quantities without the use of horseshoe crab. By doing so, we will manage to preserve the horseshoe crab’s vulnerable population [10]. Research related to genetic engineering of horseshoe crab has been carried out in many countries, but in Indonesia, it has not been carried out, especially for factor C from *T. gigas* species.

*Tachypleus gigas* is a horseshoe crab found in the Asia Pacific. Although definitive data are lacking [11], in fact *T. gigas* is found in almost every part of Indonesia, from Sumatra to Java, Kalimantan, and Maluku [12]. Horseshoe crabs found are usually discarded or caught in Indonesia and are not considered as an important fishery. Investigation into its morphology found that the telson of Indonesian horseshoe crab *Tachypleus* genus is spiny [13]. The color and spines of its ophistoma distinguish *T. gigas* and *Tachypleus tridentatus*. *Tachypleus gigas* is blackish brown in color, whereas *T. tridentatus* is greenish brown. This study aimed to produce amebocyte lysate from *T. gigas* and test its ability through TAL assay and TAL activity. We focused on the identification of the factor C cDNA molecule and the amplification of *T. gigas* amebocytes. Identification of the factor C cDNA molecule includes several bioinformatics assessments, which are included in the preliminary studies prior to the production of recombinant proteins.

## Methods

**Fig. 1:**
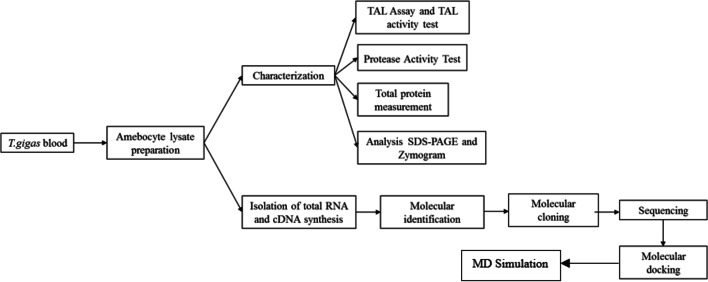
An overview of the workflow of *T. gigas* research, starting with blood sampling, followed by protein characterization (TAL assay and zymogram analysis) and RNA isolation for molecular identification, namely cloning, sequencing, molecular docking, and MD simulation

A total of 94 horseshoe crab (*T. gigas*) blood was taken from the Banyuasin Estuary, South Sumatra. *Escherichia coli* DH5α and pGEM-T Easy plasmid were used as cloning host and cloning vector, respectively. Amplification of COI and factor C genes was done using specific forward and reverse primer pairs as shown on Table 3. Animal used in this study complied guidelines and approved by Ethical Committee “Komite etik Penelitian Kedokteran dan Kesehatan (KEPKK)” Faculty of Medicine University of Sriwijaya with Protocol No. 126–2021. The workflow of this study is briefly shown in Fig. 1.

### *Tachypleus gigas* blood cell (amebocyte lysate) preparation

The cDNA was isolated from horseshoe crab blood cells (amebocytes). Blood cells were prepared by inserting horseshoe crab blood into a 3% pyrogen-free NaCl solution containing anticoagulant (in 1:1 volume ratio), following recommendations from previous studies [14, 15]. Following centrifugation at 3000 rpm for 30 min at 4 °C, the supernatant was discarded. Obtained blood cells were then stored at − 80 °C for RNA isolation. For endotoxin test, LAL activity, protease activity, and total protein measurement, blood cell was diluted in Tris–HCl 50-mM pH 8.0.

### Tachypleus amebocyte lysate (TAL) assay (gel clot method)

Endotoxin testing or TAL assay using the gel coagulation method was performed as previously described [16]. A total of 500 µL of control standard endotoxin (CSE) solution (concentration 0.0156 to 1 EU/ml) was mixed with 500 µL of horseshoe crab blood sample in a 1.5-ml pyrogen-free Eppendorf tube. The mixture was then homogenized and incubated at 37 °C ± 1 °C for 60 min ± 1 min. Horseshoe crab blood samples were tested positive for LAL if endotoxin coagulation occurred during the clotting process.

### TAL activity test

The LAL activity testing was performed based on Tinker-Kulberg method [17]. Commercial LAL (100 µl) with sensitivity of 0.25 EU/ml were gently mixed with 100 µl of standard endotoxin solution (CSE) (final concentration range: 0–50 EU/ml) in a pyrogen-free 96-well microplate and incubated at 37 °C for 1 h. The mixture was then measured at 340 nm. Another LAL assay was performed by incubating a standard endotoxin solution (50 EU/ml, with different concentrations of wild *T. gigas* amebocyte lysate (TAL) (100, 200, and 300 µg/ml).

### Protease activity test

Protease activity was conducted by following a method by Cupp-Enyard [18], with modifications. Protease test was carried out in a 96-well microplate at a wavelength of 540 nm.

### Total protein measurement

Total protein was measured using Thermo Scientific Pierce™ BCA Protein Assay Kit. Working reagent (WR) was prepared by mixing 50 parts of BCA reagent A with 1 part of BCA reagent B (50:1, reagent A:B). Sample volume at 10 µL (or BSA standard) and 200 µL of WR were added into microplate reader. Afterward, the plate was covered and incubated at 37 °C for 30 min, and then, the absorbance of mixture was measured at 540 nm.

### SDS-PAGE analysis and zymogram

Molecular mass analysis of horseshoe crab blood cell protein was carried out using SDS-PAGE electrophoresis according to a previous study [19], whereas separating and stacking gels were composed of 8% (v/v) and 4.5% (v/v), respectively, with addition of 1.5% casein. After separation process, the gel was incubated in 2.5% Triton X-100 at room temperature for 1 h, followed by incubation in 10-mM Tris HCl buffer pH 8 overnight. Gel staining was done in 0.05% Coomassie brilliant blue G-250 (Sigma) for 2–3 h. The last stage is rinsing (destaining) until clear protein bands appeared.

### RNA isolation, molecular identification, and cDNA amplification of factor C

Total RNA was isolated from horseshoe crab blood cells using a combination of TRIzol reagent (Invitrogen) and Total RNA Mini Kit (Geneaid) [20]. Total cDNA synthesis was carried out using total RNA as template (Rever Tra Ace‐α, Toyobo Co., Ltd, Japan). The cDNA of both COI gene and factor C were isolated by PCR using cDNA as template and specific primers (Table 3).

PCR was carried out for 30 cycles with denaturation conditions for 1 min at 95 °C, annealing at 63 °C for heavy chain, and 51 °C for light chain, 30 s, and then elongation at 72 °C for 2 min. Obtained factor C was then ligated into pGEMT-Easy (Promega, USA). Recombinant pGEM-T-Easy vector was introduced into *E. coli* DH5α. Colonies that were resistant to ampicillin were analyzed by colony PCR. Plasmids present in ampicillin-resistant colonies were isolated using Presto™ Mini Plasmid Kit (Geneaid). Nucleotides of cDNA factor C were then sequenced by DNA sequencing.

### Protein modeling analysis and molecular docking

BLAST analysis was carried out on www.blast.ncbi.nlm.nih.gov website. Clustering and alignment of cluster W were performed on MEGA11 software. Protein signature analysis was carried out on www.ebi.ac.uk/interpro/ website. Factor C protein sequences with FASTA file format were modeled with three web server-based protein structure models such as I-TASSER (https://zhanggroup.org/I-TASSER/), Robetta (www.robetta.bakerlab.org), and AlphaFold (https://colab.research.google.com/github/sokrypton/ColabFold/blob/main/AlphaFold2.ipynb) website.

The modeling results from the three types of web servers are compared to choose the best modeling results with several validation metrics including Ramachandran plot, ERRAT, and 3D Verify structure (www.saves.mbi.ucla.edu). Molecular docking using HDOCK was run on www.hdock.phys.hust.edu.cn website [21]. The docking parameters were computed as the default parameters of the docking online tool. The interaction between protein and ligands was visualized using Chimera 1.15 program packages and Ligplus software. Five compound ligands were retrieved from the PubChem database https://pubchem.ncbi.nlm.nih.gov/. Furthermore, the best modeling results were verified using molecular dynamics (MD) simulation for 150 ns on AMBER 20 software. The molecular docking and MD simulation protocols are similar procedures with our previous work presented in [22].

Molecular dynamics is carried out by several steps such as energy minimization, heating, equilibration, and production (run production). The minimization step was firstly performed on the system, and then, the heating step was carried for 500 ps (picosecond) to increase the temperature gradually from 0 to 300 K. The equilibration stage consists of two parts: the first for the NVT ensemble and the second for the NPT ensemble. The equilibration with the NPT ensemble was carried out for 500 ps at a constant temperature of 300 K. The next stage was the production run simulation process to see the free movement of molecules without obstacles. This stage was run in an NPT ensemble (constant number of particles, pressure, and temperature) for 150 ns with a constant temperature of 300 K.

## Results

### Endotoxin (TAL) test, TAL activity, and protease activity

TAL gel clot test is the most simple endotoxin qualitative test, which is performed by gel clot method. It shows whether the endotoxin concentration is equal, above or below the LAL reagent sensitivity. Samples were marked as positive (if the concentration is found equal or above the reagent) and negative (below the reagent concentration) results. In this study, amebocyte lysate from *T. gigas* of TAL 35 sample was able to detect endotoxin in a wide range of different endotoxin concentrations (0.0156–1 EU/ml). In contrast, TAL 36 could only detect endotoxin within the range of 0.0625 to 1 EU/ml (Table 1) endotoxin concentration.

**Table 1:** Compiled results of TAL assay of *T. gigas* amebocyte lysate of CSE concentration and its application on drinking waters

Sample of TAL	Sex	Concentration of CSE (EU/ml)							Application		
		1	0.5	0.25	0.125	0.0625	0.0312	0.0156	Water	Whole milk	Pasteurized milk
35	Male	+ +	+ +	+ +	+ +	+ +	+ +	+ +	+ +	+ +	-
36	Female	+ +	+ +	+ +	+ +	+ +	-	-	-	-	-

Sample TAL 35 showed positive results in detecting endotoxin in water and whole milk, but not in pasteurized milk. On the other hand, TAL 36 showed negative results for endotoxin detection in water, whole milk, and pasteurized milk.

**Fig. 2:**
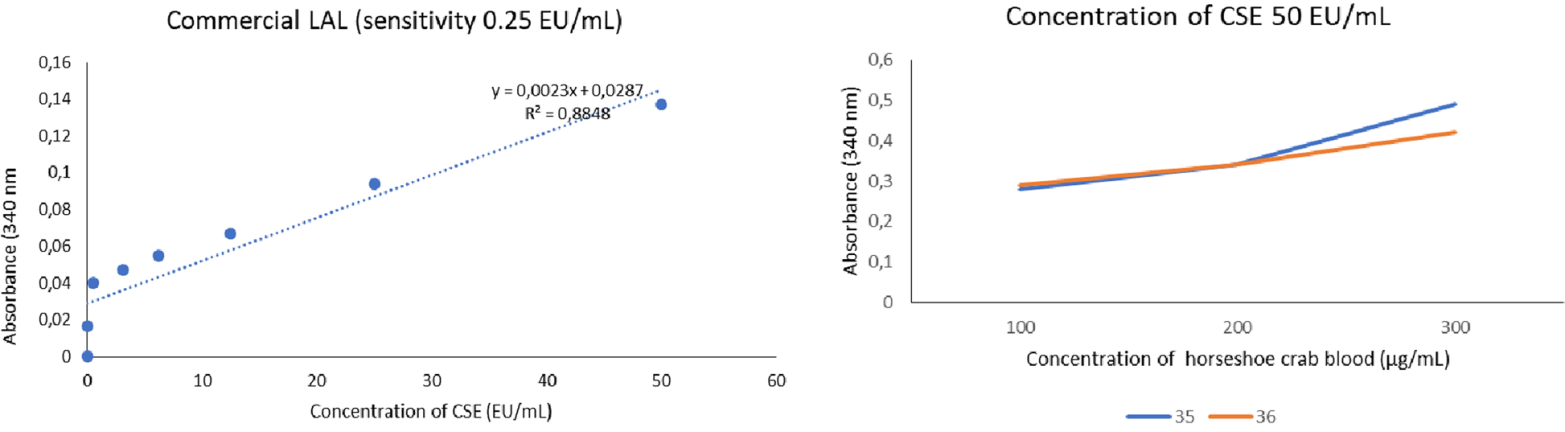
The LAL commercial activity in different concentrations of CSE **A** and TAL activity in CSE concentration of 50 EU/ml **B**

Higher endotoxin concentrations led to higher LAL activity, as shown in Fig. 2. This is in line with the study of Tinker-Kulberg et al. [17]. In addition, higher LAL reagent concentration promotes more active LAL reaction. The LAL sensitivity ranges from 0.05 to 50.0 AU/ml. This range belongs to the LAL-specific character which is useful for detecting LPS in human blood because up to 80% of pathogens cause sepsis in general [17].

**Fig. 3:**
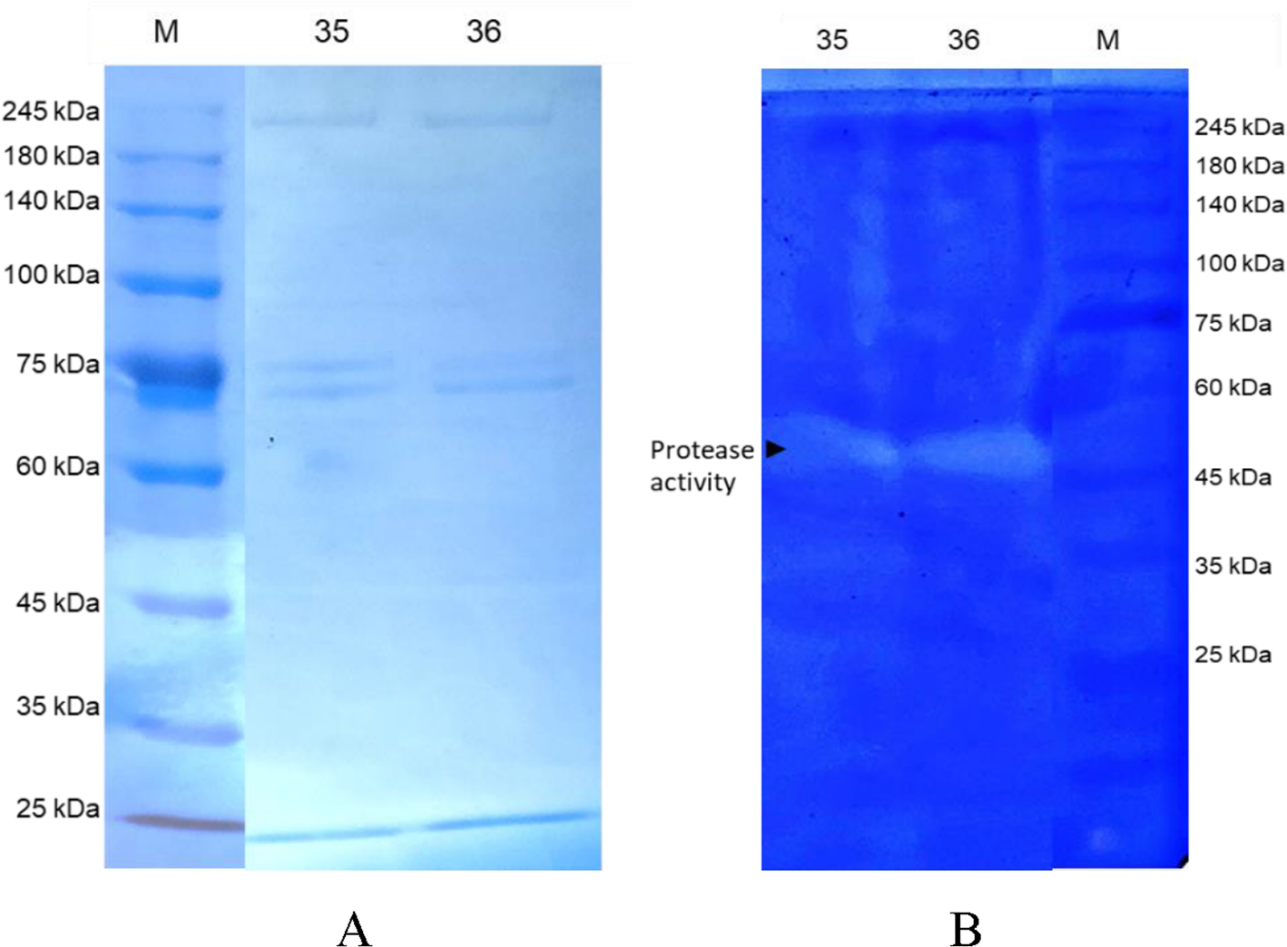
Results of SDS-PAGE **A** and casein zymogram **B** of protein from blood of *T. gigas*. On both samples (35 and 36), three protein bands spotted on acrylamide gel of SDS-PAGE on 245, 75, and 24 kDa. However, zymogram reveals proteolytic activity of protein substance on molecular weight between 45 and 60 kDa, which is considered as factor C light chain

Table 2 shows sample 36 had higher total protein value and protease activity than sample 35. Body weight, sex, body mechanisms, and environment can all have an impact on total protein content of TAL samples. Furthermore, protease activity of TAL is influenced by incubation time, temperature, solution pH, and inhibitor. The protease activity of *T. gigas* amebocyte can also be seen in zymogram analysis shown in Fig. 3.

**Table 2:** Protease activity and total protein of *T. gigas* blood

Sample code	Protease activity (U/ml)	Total protein (µg/ml)
35	367	1021
36	430	1765

Based on Fig. 3, molecular masses of horseshoe crab amebocytes are approximately 245, 75, and 25 kDa. The zymogram analysis reveals protease activity in amebocyte proteins with molecular masses less than 60 kDa. This protein is thought as a factor C light chain of large granules.

### Molecular identification of horseshoe crab

**Fig. 4:**
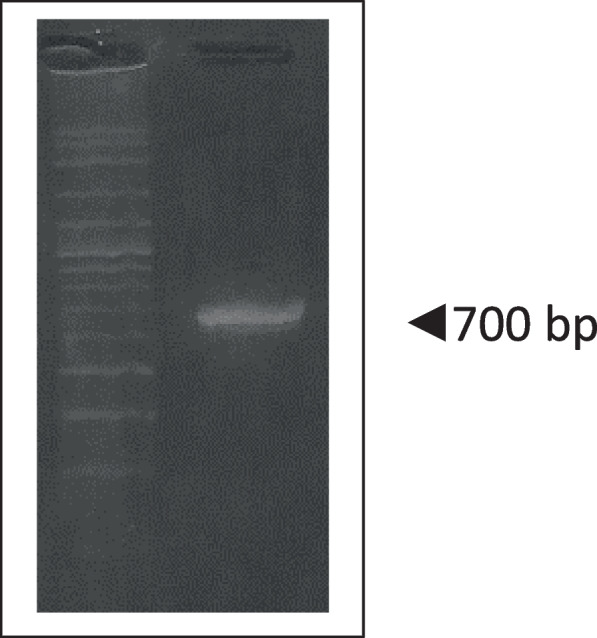
Amplification of horseshoe crab’s COI gene. The amplicon spotted at 700 bp

The cytochrome c oxidase subunit 1 (cox1 or COI) gene in mitochondria is commonly used to identify animal species using DNA markers. The cDNA of COI gene to identify horseshoe crab blood sample had been amplified by PCR (Fig. 4).

Species identification out of nucleotide sequences of these COI genes was compared to COI genes in NCBI using BLAST. The BLAST analysis revealed that COI gene of horseshoe crab blood sample had 98.68% identity similarity to mitochondrial genome of *T. gigas* with accession number NC 052,701, with 49% query coverage.

### cDNA cloning of factor C

**Fig. 5:**
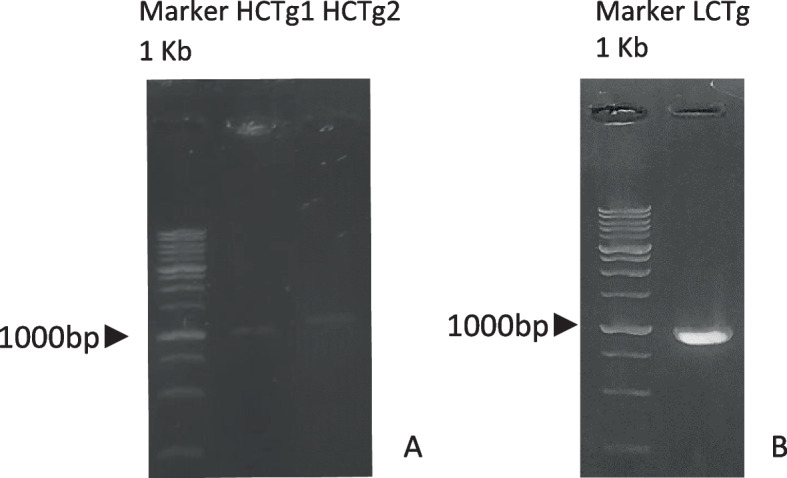
The results of the isolation of cDNA fragments. **A** cDNA fragments encoding heavy chain factor C from *T. gigas* (HCTg). **B** cDNA fragment encoding the *T. gigas* factor C light chain factor (LCTg)

Factor C consists of two glycoprotein chains, namely heavy and light chains. The heavy chain itself (HCTg) encodes cDNA that consists of two overlapping fragments. Isolation of heavy chain factor C of *T. gigas* (HCTg) using spesific primers (Table 3) presents protein size of 1046 bp for fragment 1 (HCTg1) and 1108 bp for fragment 2 (HCTg2) (Fig. 5A). On the other hand, isolation of light chain factor C (LCTg) using specific primers (Table 3) presents protein in a size of 990 bp (Fig. 5B). The heavy chain and light chain were then ligated to pGEM-T-Easy and inserted into *E. coli* DH5α. Plasmid DNA had been isolated from *E. coli* putative transformant and considered resistant to antibiotics.

**Table 3:** Specific primer pairs that were used to amplify cDNA from *T. gigas* blood

Fragments	**Primer pairs**	
	**Forward**	**Reverse**
COI gene	GGTCAACAAATCATAAAGATATTGG	TAAACTTCAGGGTGACCAAAAAATCA
Heavy chain 1	ATGGTCTTAGCGTCGTTTTTGGT	TGGATCCTGACAGGTTCACCAACATCAT
Heavy chain 2	TGTGTTAAAGTGGCAGACAGAGAGG	GGGTTGTGAGGATCTAGGTTTTGGAGGA
Light chain	TCCTCACAACCCTCTACT	TCAAATGAACTGCCTAATCC

**Fig. 6:**
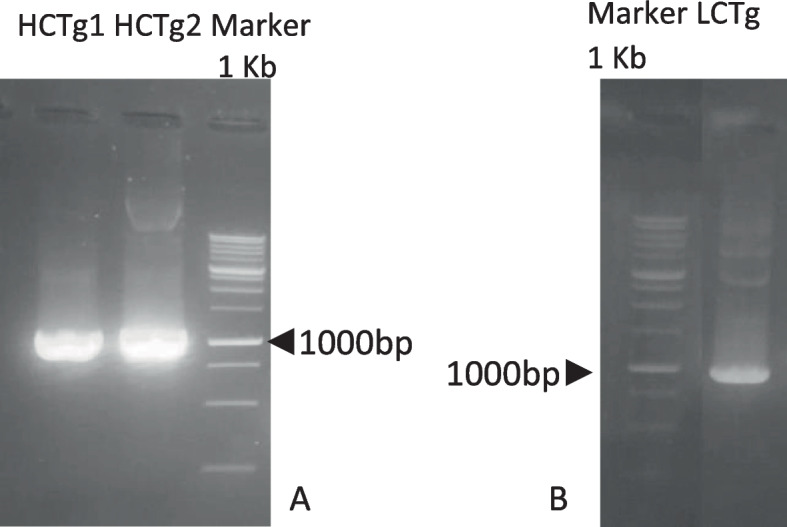
**A** Amplification results of HCTg in recombinant plasmid pGEM-T-Easy-HCTg and **B** LCTg found in recombinant plasmid pGEMT-easy-LCTg (**B**)

Amplified DNA by PCR reaction confirms that HCTg and LCTg had been successfully inserted into pGEM-T-Easy plasmid and forms recombinant plasmid (Fig. 6).

Based on the BLAST results, the sequence of *T. gigas* factor C has similarities with existing C factor of *T. tridentatus* with 96.86% identity similarity and 100% query coverage. When the *C. rotundicauda* factor was compared with *Limulus polyphemus*, the identity and query coverage percentages were recorded at 96.50% and 100% for *C. rotundicauda* and 90.29% and 34% for *L. polyphemus*, respectively (Table 4).

**Table 4:** BLAST result of sequence of *T. gigas* factor C

Species	Query cover	Percent identity	Accession number	Reference
*Tachypleus tridentatus*	100%	96.86%	*P28175.1*	Muta et al. [23]
*Carcinoscorpius rotundicauda*	100%	96.57%	*Q26422.1*	Ding et al. [24]
*Tachypleus tridentatus*	100%	96.57%	*AAL75577.1*	Wang et al. [25]
*Carcinoscorpius rotundicauda*	99%	96.45%	*AAB34362.1*	Ding et al. [24]
*Tachypleus tridentatus*	63%	94.91%	*BAA14316.1*	Muta et al. [23]
*Limulus polyphemus*	24%	90.00%	*XP_013792128.1*	From a genomic sequence (*NW_013675215.1*) annotation
*Limulus polyphemus*	22%	91.77%	*XP_013793100.1*	from a genomic sequence (*NW_013678219.1*) annotation

**Fig. 7:**
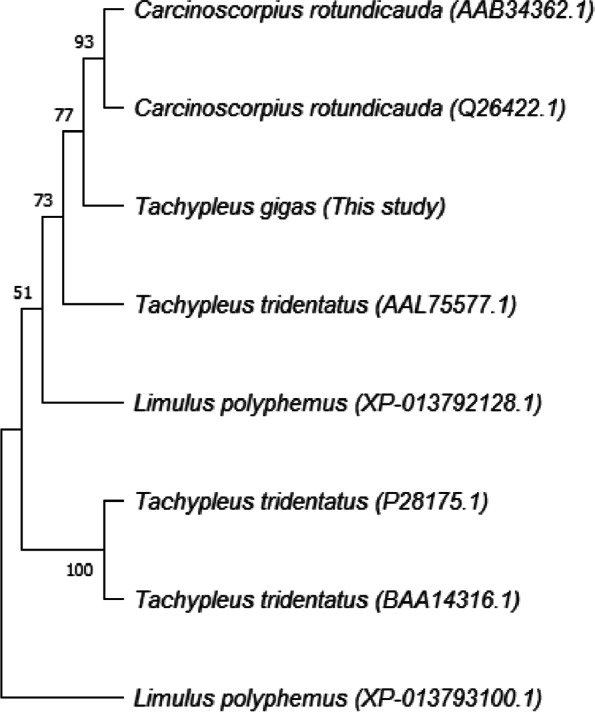
Dendrogram clustering factor C of several species of horseshoe crab

Alignment of factor C amino acids from *T. gigas* with factor C of Asian horseshoe crab species, namely *T. tridentatus* and *C. rotundicauda*, was carried out using ClustalW alignment method using MEGA 11. As shown in Fig. 7, the factor C of *T. gigas* is distinct from factor C of *L. polyphemus* and similar to factors C of *T. tridentatus* and *C. rotundicauda* (Asian horseshoe crab).

Amino acid alignment results confirmed that amino acid sequences of factor C of those three species of horseshoe crab are similar. Heavy chain amino acids of T*. gigas* have many differences compared to heavy chains of *T. tridentatus* and *C. rotundicauda*, while the light chain *T. gigas* tends to have more similarities compared to light chains of T*. tridentatus* and *C. rotundicauda*. At the 874th position, the light chain amino acid of *T. gigas* is serine, while the other light chain amino acid is proline.

**Fig. 8:**
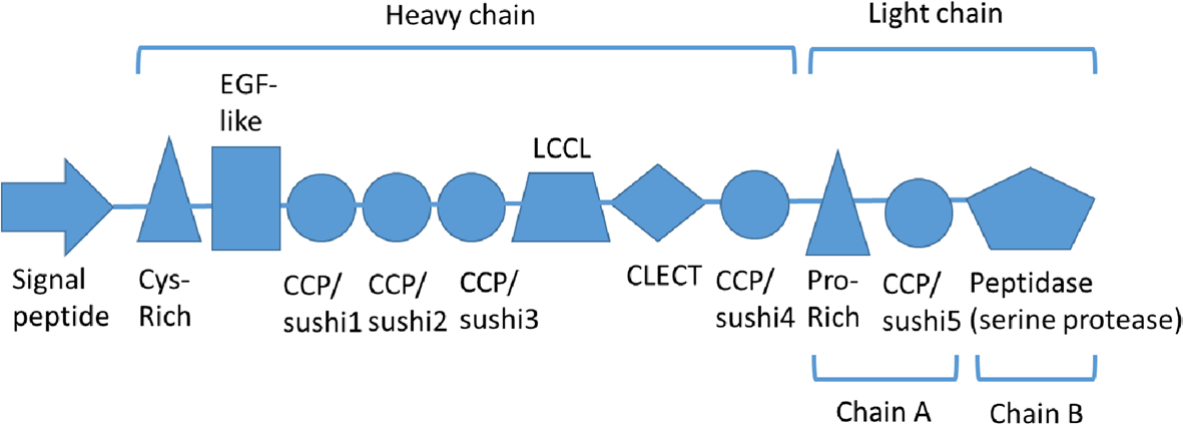
Summary of factor C domain structure

**Fig. 9:**
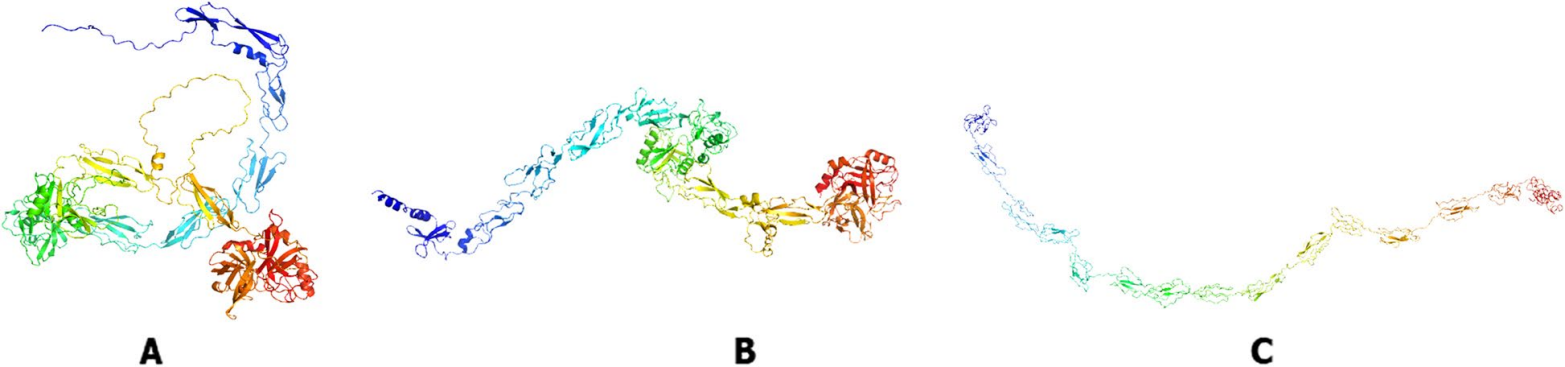
3D structure model of *T. gigas* factor C created by **A** AlphaFold, **B** Robetta, and **C** I-TASSER

Identification of *T. gigas* protein classification was carried out by testing the characteristics of the domain and amino acid sequences. The factor C protein signature was analyzed via InterPro European Bioinformatics Institute (EBI) web severs [26]. Results showed that factor C of *T. gigas* consists of a signal peptide and two glycoprotein chains, heavy chain and light chain. The signal peptides are located at amino acid positions 1–25, which consist of N-region signal peptide (1–5), H-region signal peptide (6–17), and C-region signal peptide (18–25). On the other hand, heavy chain has cysteine-rich regions (Cys-rich region), four complement control proteins (CCP), EGF (epidermal growth factor)-like, LCCL, and C-type lectin (CLECT). These CCPs or sushi domains include amino acid positions 142–196 (CCP1), 199–255 (CCP2), 260–322 (CCP3), and 576–635 (CCP4). The EGF like is located at amino acid positions 125–136. The LCCL domain is situated at positions 325–421, and CLECT is at amino acid positions 436–565. Light chain is one fragment of factor C, which belong to serine protease that plays a role in coagulin system in horseshoe blood. Protein signatures analysis found that light chain has a proline-rich region (Pro-rich region), CCP5 located at amino acid position 702–749, and a trypsin-like serine protease domain at position of 762–1019, which belongs to peptidase family S1 (Figs. 8 and 9). The domain Factor C of T. *gigas* was analyzed using the European Bioinformatics Institute's (EBI) Inter Pro web server shown in Fig. 8.

 The Factor C protein was modeled with three web servers including I-Tasser [27], Robetta [28] and AlphaFold [29] website. Then, the results of 3D structure of proteins were selected based on the several validation analysis such as Ramachandran plot, Errat and 3D verify Structure. The 3D structure of each protein modeling is shown in Fig. 9.

**Fig. 10:**
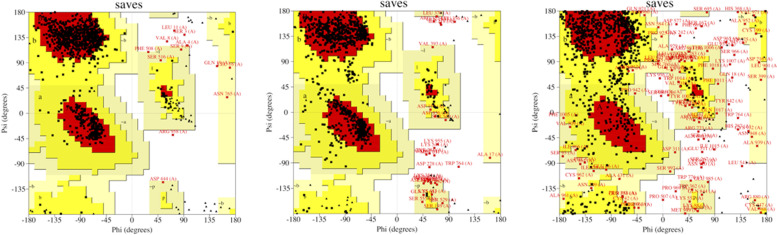
Ramachandran plot analysis of *T. gigas* factor C. **A** Model by AlphaFold, **B** model by Robetta, and **C** model by I-TASSER

Ramachandran plot is one of the most central concepts in structural biology; it is commonly used to verify the modeling of protein structure. In this study, Ramachandran plot analysis of factor C protein structure of *T. gigas* is shown on Fig. 10.

Ramachandran plot analysis shows that factor C structure model with three modeling website (Fig. 10) has different values. The AlphaFold modeling showed the best Ramachandran results compared to other models because it has a residue in the most favorable region of 88.3% (quadrant 1), a residue in allowable additional region of 10.3% (quadrant 2), a residue in generously permitted region of 0.7% (quadrant 3), and the residue in the area that is not permitted is 0.7% (quadrant 4). Greater amino acid residues percentage in the most preferred region, and lower residue in not permitted region, relates to better quality and stability of protein structure [30]. Results analyses show that *T. gigas* factor C structure with AlphaFold modeling has medium quality and tends to be stable. Protein structure quality is determined good if it has a preferred area score of > 90% and < 2% score for not allowed area [31].

**Fig. 11:**
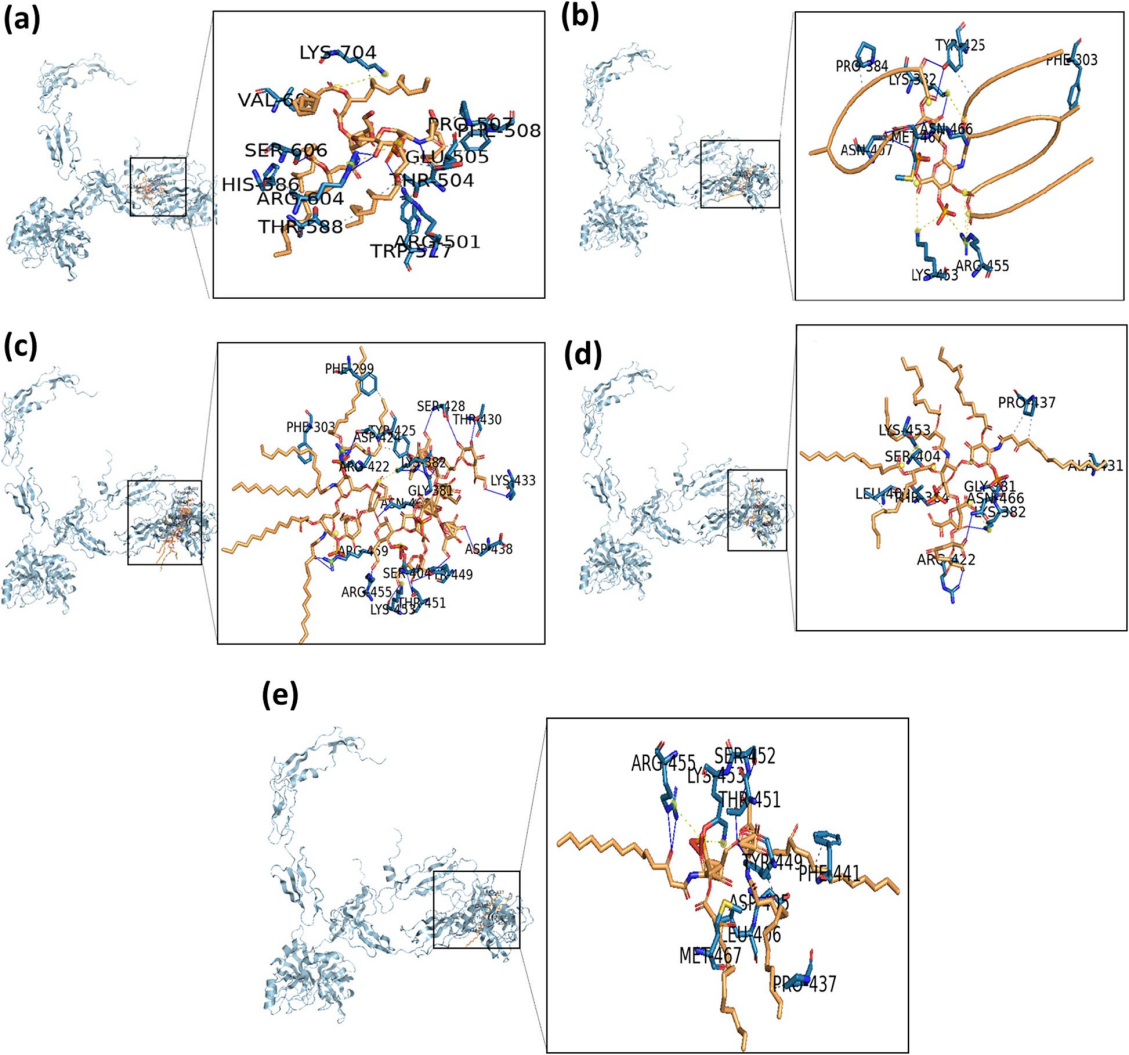
Binding of the ligand to protein factor C receptor site. **A** Complex 1 (lipid A ligand), **B** complex 2 (diphosphoryl lipid A ligand), **C** complex 3 (core lipid A ligand), **D** complex 4 (Kdo2 lipid A ligand), and **E** complex 5 (disaccharide lipid A ligand). The conformational poses of each complex were visualized with the Chimera program

High-resolution structures generally have an ERRAT value of more than 95% and a Verify 3D value of more than 80%. Better scores on ERRAT and Verify 3D, which is referred as a “overall quality factor” for nonbonded atomic interactions, indicate higher level of quality [32]. Table 5 listed the ERRAT and Verify 3D score for the 3D structures of factor C protein obtained from AlphaFold, Robetta, and I-TASSER. From this table we found that the 3D model from AlphaFold is better score than other models in which the value of the ERRAT and Verify 3D are 87.053% and 81.26%, respectively. Thus, the created model from AlphaFold webserver is used to continue molecular docking with some of the ligands selected in (Table 6, Fig. 11).

**Table 5:** Quality evaluation of factor C protein structure with ERRAT and Verity 3D web server

Modeling structure of factor C protein	ERRAT (%)	Verify 3D (%)
AlphaFold	87.05	81,26
Robetta	85.42	87,63
I-TASSER	59,546	49,46

**Table 6:**
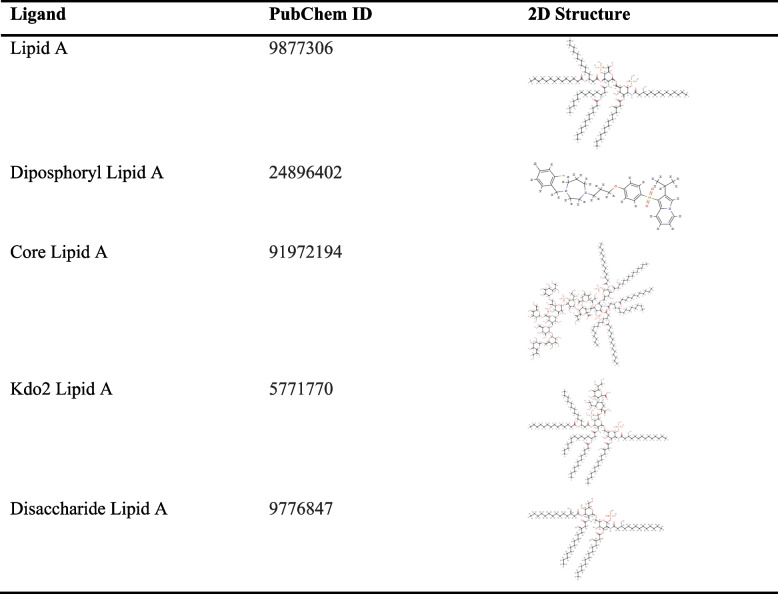
The list of ligands from lipid A

Molecular docking is a well-known method to design a new drug using computational approaches. This method was used to identify the interaction between a small molecule (ligand) and protein target (receptor). In this research, lipopolysaccharide (lipid A) and factor C are utilized as a ligand and a receptor, respectively. LPS is a fraction that is on the outer membrane of gram-negative bacteria, which causes toxic effects such as fever, septic shock, multiorgan failure, and even death. LPS has a tripartite structure consisting of three covalently linked domains, namely the O-antigen-specific chain, core polysaccharide, and lipid A. The proinflammatory bioactivity of LPS resides in the most structurally conserved glucosamine-based phospholipid known as lipid A.

**Fig. 12:**
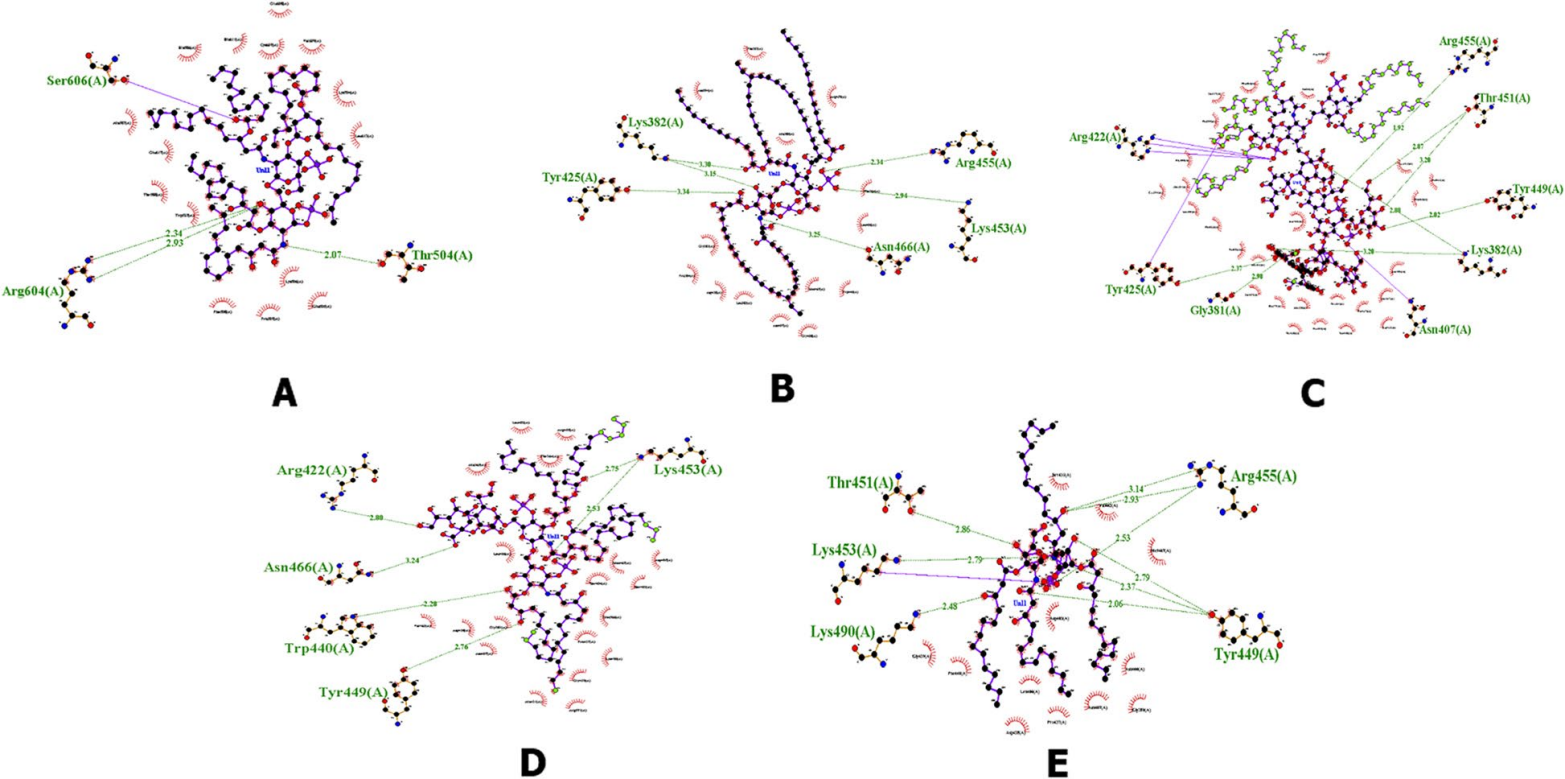
Visualization of ligand binding to the protein factor C receptor. **A** complex 1, **B** complex 2, **C** complex 3, **D** complex 4, and **E** complex 5

Docking simulation with five complex ligands with various bonds, which are visualized in Fig. 12. All ligands can bind to the receptor, as indicated by the docking score that is negative. These results indicate that all ligands can form complexes with receptors. It can be seen that the top three complexes, namely complex 2, complex 3, and complex 4 (Table 7), showed higher docking scores with values of − 302.86, − 467.93, and − 322.98, respectively, and the confidence scores for each docking result were 0.9551, 0.9983, and 0.9695.

**Table 7:** Docking results using the HDOCK website between protein factor C and lipid A

Complex	Docking score	Confidence score
Complex 1 (FC-lipid A)	− 246.43	0.8731
Complex 2 (FC-diposphoryl lipid A)	− 302.86	0.9551
Complex 3 (FC-core lipid A)	− 467.93	0.9983
Complex 4 (FC-Kdo2 lipid A)	− 322.98	0.9695
Complex 5 (FC-disaccharide lipid A)	− 269.43	0.9160

**Fig. 13:**
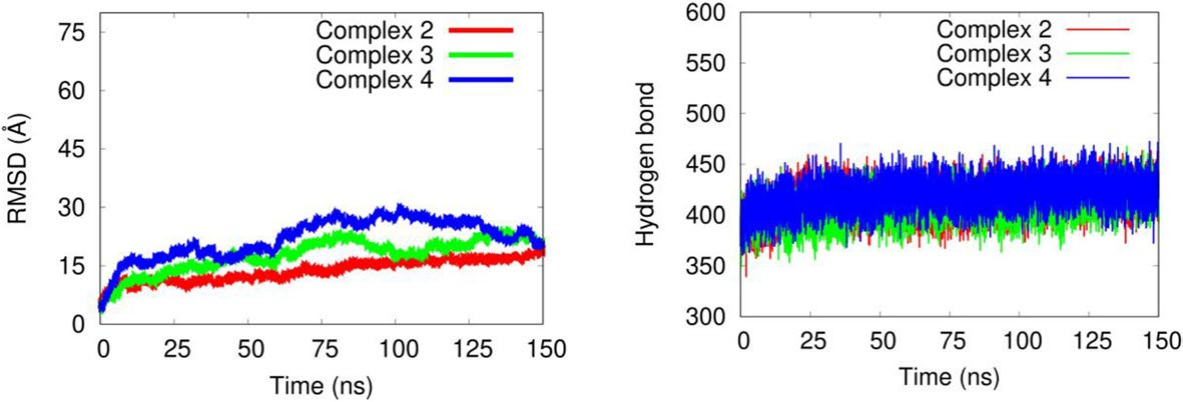
**A** RMSD value of the complexes, **B** Hydrogen bond from MD trajectory. The complexes 2, 3, and 4 are presented by red, green, and blue colors, respectively

The protein stability exhibited some changes, which were evaluated using the root-mean-square deviation (RMSD) and hydrogen bonds of the protein–ligand complex (Fig. 13).

## Discussion

The LAL assay is performed by a reaction between LPS and zymogen factor C that initiates a protease cascade response. Factor C induces factor B, which transforms proclotting enzymes to clotting enzymes. Furthermore, to generate a coagulin gel, the two peptide bonds in coagulogen will be catalytically broken down [33]. However, the LAL test may also show some uncertainties, when it comes to measurement results [34]. For example, if LAL reagent has a sensitivity of 0.125 EU/ml, the endotoxin test must coagulate endotoxin in a concentration of 0.25 EU/ml. However, even if the reagent may coagulate at 0.125 EU/ml, it may not coagulate at a concentration of 0.06 EU/ml or 0.03 EU/ml.

Our sample proves diverse reading results on their endotoxin tests. The TAL 35 sample detected endotoxin in different ranges of concentration compared to TAL 36. Those different results of LAL reading between samples might be due to some factors that may be involved during the process, such as the presence of sample impurities, along with sample conditions such as pH and temperature — all of those factors are able to interfere the cascade reaction and reduce sensitivity and specificity of LAL testing [35]. Other findings also mentioned some other factors that affect LAL assay, such as chemical inhibitors that cause chelation of divalent cations required for LAL reactions, protein denaturation, dilution of chelation effect, calcium (Ca) replacement in anticoagulants, and even changes in LAL supplier [1].

While both samples were tested on consumable drinks, variable results were also obtained. However, apart from all the factors that could contribute to the endotoxin test results in the tested consumption drinks as described in previous studies, we also considered the amount limit of endotoxin permitted by the FDA. Tolerable endotoxin limit for drugs and food that parenterally administered substances is five endotoxin units (EU) per kg body mass. Based on the calculation, endotoxin amount at 350 EU on a body mass of 70 kg per person or a 350 EU/ml tolerance for parenteral products where a single dose consists of 1 ml is allowed. No limitations are currently available for endotoxin present in orally administered products [36].

Horseshoe crab has two types of granular molecular masses, which are divided into two types: large (up to 1.5 m in diameter) and small (0.6 m in diameter). The large granules contain more than 25 proteins, and most of them have molecular masses ranging from 8 to 123 kDa. Clotting factors B, C, and G, as well as proclotting and antimicrobial (anti-LPS) factors, are present in large granules. Small granules, on the other hand, contain at least six proteins with molecular masses less than 30 kDa. Other antimicrobial substances are present in these granules [37].

Zymogram analysis showed the presence of protease activity in molecular masses less than 60 kDa. This protein is thought as a factor C light chain of large granules. Factor C has a molecular mass of 123 kDa and is composed of two glycoprotein chains, heavy chain (80 kDa) and light chain (43 kDa) in *T. tridentatus* [31] and heavy chain (80 kDa) and light chain (52 kDa) in *C. rotundicauda* [38].

Factor C consists of two glycoprotein chains, namely heavy and light chains, where the heavy chain itself (HCTg) encodes cDNA that consists of two overlapping fragments. Furthermore, both of those heavy and light chains had been isolated from *T. gigas* blood using a specific primer (Fig. 6 A and B). Alignment of factor C nucleotides sample of *T. gigas* on NCBI with BLAST showed that factor C similar to factor C derived from other horseshoe crab species such as *L. polyphemus* (data taken from NCBI with accession number: XM_013937646), *T. tridentatus* (D90271.1), and *C. rotundicauda* (S77063.1).

The LPS binding site is present in the N-terminal cysteine-rich region of the molecule and contains a tripeptide sequence consisting of an aromatic residue flanked by two conserved basic residues in other mammalian LPS-recognition proteins [39]. As binding site is important for LPS sensitivity, there are two critical factors for successful binding of factor C to LPS: (1) the presence of multiple binding sites for LPS on factor molecule and (2) high positive cooperativeness in LPS binding [40].

Protein structure is formed by condensed amino acid sequences via peptide bonds into the universe conformation. Ramachandran plots are widely used to assess the stereochemical quality of structures solved by experimental techniques as well as protein models built by predictive methods by analyzing dihedral angles in allowed or not allowed regions [41]. The Ramachandran plot’s allowable area indicates that when peptide atoms are given a standard radius, they do not collide. Additional regions, sometimes referred as bridge regions (because they connect the alpha and beta regions), become permissible if the atoms are given a smaller radius that represents smallest value that is considered reasonable [42]. The forbidden region is the conformation in which protein atoms are closer together than the sum of atoms’ van der Waals radii. This region is forbidden for all amino acids with side chains (except glycine, which has no side chains) [43].

Molecular docking is used to investigate the small molecule behavior inside the binding pocket of protein and to predict the affinity that might be occurred during binding [44]. From our docking simulation, we suggested that three ligands, i.e., diposphoryl lipid A, FC-core lipid A, and Kdo2 lipid A, can be activators of the factor C protein by binding to the region of the receptor to form a ligand-receptor complex. Therefore, three structures (complexes 2, 3, and 4) were selected for further analysis [45].

To confirm the stable structure of the three complexes, all-atom molecular dynamics simulation (MD) was carried out in water solvent. We then analyzed the root-mean-square deviation (RMSD) and hydrogen bonds of those complexes along the simulation. RMSD value as a function of evolution time estimated from the trajectories of MD simulations is shown in Fig. 13a. From this figure, all complexes involved fluctuations during the simulation. The fluctuation along simulation can be influenced by atomics interactions in the system via electrostatic interaction and hydrogen and hydrophobic interactions which involve structural rearrangement at the protein and ligand molecule interface [22, 45]. Moreover, the factor C protein has a large structure (1019 amino acids) with several domains placing different regions which make the protein structure tend to fluctuate along the MD simulation. Additionally, the hydrogen bond formation of each complex was also analyzed. This formation involves a crucial function in supporting the rigidity of a biomolecular complex. Figure 13b showed the hydrogen profiles for all complexes along the simulation, indicating the hydrogen bond formation contributed in keeping a stable complex during the simulation process.

## Conclusions

The molecular identification using COI gene revealed the horseshoe crab blood had 98.68% identity similar to mitochondrial genome of *T. gigas*. The sequence of cDNA cloning of factor C revealed that the gene encoding factor C of *T. gigas* was 97.03% similar to *T. tridentatus*. The endotoxin test showed that TAL 35 had endotoxin sensitivity in a range of 0.0156–1 EU/ml, while TAL 36 had a sensitivity between 0.0625 and 1 EU/ml. *T. gigas* amebocytes have protease activity in molecular mass sizes less than 60 kDa, with 367 U/ml for TAL 35 and 430 U/ml for TAL 36. This protein is thought as a factor C light chain. The docking evaluated lipopolysaccharide (lipid A) and factor C as a ligand and a receptor, respectively. The results suggested that three ligands, i.e., complex 2 (diphosphoryl lipid A), complex 3 (core lipid A), and complex 4 (Kdo2 lipid A) can be activators of the factor C protein by binding to the region of the receptor to form a ligand-receptor complex. Based on our results, the unique ability of *T. gigas* amebocyte lysates to detect endotoxins and biotechnologically engineered of recombinant Factor C (rFC) from *T. gigas* species in Indonesia is possible for further medical purposes and simultaneously preserving the horseshoe crab’s vulnerable population.

## Data Availability

Not applicable.

## Abbreviations

TAL: *Tachypleus* Amebocyte lysates
MD: Molecular dynamic
LPS: Lipopolysaccharides
DIC: Disseminated intravascular coagulation
ARDS: Adult respiratory distress syndrome
rFC: Recombinant Factor C
CSE: Control standard endotoxin
RMSD: Root-mean-square deviation
